# Biological Age Prediction From Wearable Device Movement Data Identifies Nutritional and Pharmacological Interventions for Healthy Aging

**DOI:** 10.3389/fragi.2021.708680

**Published:** 2021-07-15

**Authors:** Rebecca L. McIntyre, Mizanur Rahman, Siva A. Vanapalli, Riekelt H. Houtkooper, Georges E. Janssens

**Affiliations:** ^1^ Laboratory Genetic Metabolic Diseases, Amsterdam Gastroenterology, Endocrinology, and Metabolism, Amsterdam Cardiovascular Sciences, Amsterdam UMC, University of Amsterdam, Amsterdam, Netherlands; ^2^ Department of Chemical Engineering, Texas Tech University, Lubbock, TX, United States; ^3^ NemaLife Inc., Lubbock, TX, United States

**Keywords:** aging, machine learning, NHANES, wearable device, doxazosin, biological age

## Abstract

Intervening in aging processes is hypothesized to extend healthy years of life and treat age-related disease, thereby providing great benefit to society. However, the ability to measure the biological aging process in individuals, which is necessary to test for efficacy of these interventions, remains largely inaccessible to the general public. Here we used NHANES physical activity accelerometer data from a wearable device and machine-learning algorithms to derive biological age predictions for individuals based on their movement patterns. We found that accelerated biological aging from our “MoveAge” predictor is associated with higher all-cause mortality. We further searched for nutritional or pharmacological compounds that associate with decelerated aging according to our model. A number of nutritional components peak in their association to decelerated aging later in life, including fiber, magnesium, and vitamin E. We additionally identified one FDA-approved drug associated with decelerated biological aging: the alpha-blocker doxazosin. We show that doxazosin extends healthspan and lifespan in *C. elegans*. Our work demonstrates how a biological aging score based on relative mobility can be accessible to the wider public and can potentially be used to identify and determine efficacy of geroprotective interventions.

## Introduction

**FIGURE 1:**
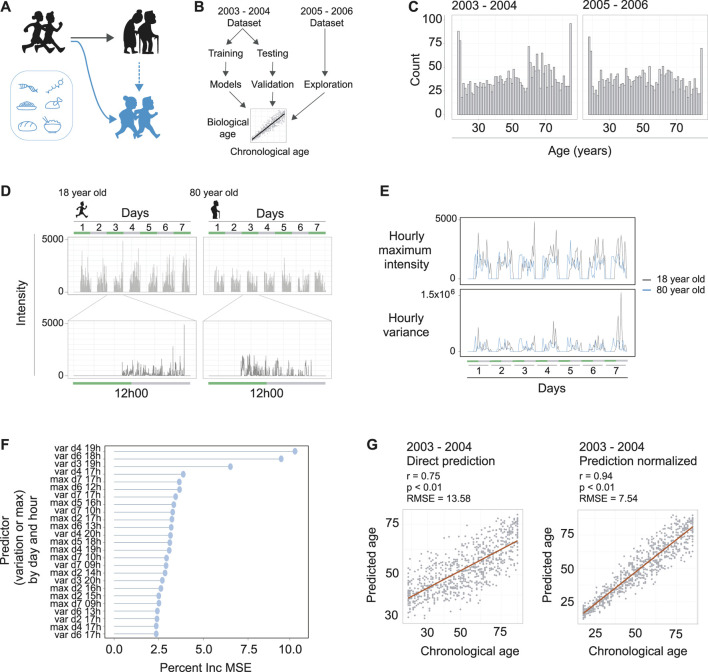
Machine learning to predict age from wearable device movement data. **(A)** Schematic representing the typical aging process (black figures), accompanied by reduced locomotive capacity. Genetics, drugs, and nutrition are able to promote healthy aging, or, in the case of drugs and nutrition, can promote healthy aging and thereby improved locomotive capacity (blue figures). **(B)** Schematic showing machine learning strategy to build age predictors from wearable devices that measure accelerometer readings. The two publically available NHANES datasets that included accelerometer data were used. The 2003–2004 dataset was used for model building and validation. The 2005–2006 dataset was used for validation and exploration of associations with accelerated or decelerated biological aging. **(C)** The distribution of ages and total counts of individuals for each dataset, following data quality filtering steps. Left panel is the 2003–2004 dataset and right panel is the 2005–2006 dataset. **(D)** Example of accelerometer readings in the data for a typical 18 year old **(left hand panels)** and typical 80 year old **(right hand panels)**. The data covers seven days of readings **(top panels)**, and a single day contains readings from the moment the individual attached the device in the morning until they removed it in the evening **(bottom panels)**. **(E)** Example of hourly maximum intensity **(top panels)** and hourly variance **(bottom panels)** for the 18 and 80 year olds depicted in panel D. The maximum intensity and variance of readings per hour over the seven days were used as input for machine learning to predict age of the individual. **(F)** The strength of each predictor (variance or maximum intensity), by day and hour in the dataset, for the random forest machine learning model. Strength of each predictor is interpreted from the models percent increase of mean standard error (Percent Inc. MSE) calculated for each predictor. **(G)** The prediction of the models for the validation dataset. **Left panel** is direct prediction on the raw accelerometer data form the validation dataset, with *r* = 0.75, *p* < 0.01, and an RMSE of 13.58 years. **Right panel** is the normalization of the prediction based on a priori knowledge of the participant’s ages, resulting in *r* = 0.94, *p* < 0.01, and an RMSE of 7.54 years

The aging human population and associated increase in age-related diseases present a growing burden to the healthcare system and society (Partridge et al., 2018). Genetics, dietary factors, and pharmaceuticals can all be considered geroprotective. These can potentially decelerate the aging processes, and thereby the progression of age-related disease (Figure 1A). However, variation in rates of aging in humans, and the ethical, practical and financial limitations to perform human lifespan studies, make it difficult to translate fundamental findings into clinical practice (Partridge et al., 2018). Therefore, the ability to predict biological age as a measure of health and longevity is an attractive prospect.

A variety of biological age predictors have been generated already, using parameters such as telomere length, gene expression profiles, or metabolomics (reviewed by Jylhävä et al., 2017). A composite biomarker predictor has also been developed, utilizing 18 biomarkers over multiple organ systems in young adults (Belsky et al., 2015). Perhaps the best described “aging clock” is based on epigenetic age *via* DNA methylation (Bocklandt et al., 2012; Hannum et al., 2013; Horvath, 2013). DNA methylation clocks have then been used to assess behavioral lifestyle factors for their effect on biological age (Quach et al., 2017). This allowed factors such as diet, exercise and intake of pharmaceuticals to be evaluated for their influence on longevity. These existing age predictors have added great value to the field, yet most require blood or tissue samples and are therefore inaccessible to the general public.

Arguably the most downstream measure of healthy aging is one’s ability to move. Mobility translates to independence in the ability to care for one’s self and therefore quality of life. In addition, movement can be considered the culmination of many molecular and physiological processes of aging summed together. The popularity of wearable movement tracker devices provides accessible and accurate movement data for a large portion of the population. Movement parameters from a wearable device have been associated with mortality (Chudasama et al., 2019), as well as biological aging to predict frailty, risk of chronic disease, and mortality (Pyrkov et al., 2018a; Pyrkov et al., 2018b; Rahman and Adjeroh, 2019).

Here, using publicly available NHANES data we develop a predictive model for biological age based on movement measured by a wearable device. We then used this model to identify and assess efficacy of longevity interventions. Namely, we identify nutritional components associated with healthy aging, as well as a pharmaceutical drug; doxazosin. Finally, we confirm that doxazosin causally influences longevity and promotes greater movement later in life *via* lifespan and healthspan measurements in the worm *C. elegans*.

## Methods

### Dataset Downloading and Processing

NHANES data from 2003–2004 and 2005–2006 were used in this study: https://wwwn.cdc.gov/nchs/nhanes/. Accelerometer data was available in the PAXRAW_C and PAXRAW_D files, and the demographic data was available in DEMO_C and DEMO_D files. The nutritional data was accessed using the DR1TOT_D file and use of prescription drugs were accessed with the RXQ_RX_D file. For use in model building, the 2003–2004 accelerometer data was downloaded from the NHANES repository, filtered to ensure that data was present for each minute of the seven days, devices were calibrated, daily data contained sufficient accelerometer entries (10% data >0), and participants were aged 18 and older. This resulted in high quality dataset of 7 days of accelerometer data for 2,634 adults. For use in model validation, the 2005–2006 NHANES accelerometer data was preprocessed identically and resulted in data for 2,505 adults. Linked mortality data on the participants from NHANES was accessed at the link below. See supplementary materials for extended methods on data access and processing.

ftp://ftp.cdc.gov/pub/Health_Statistics/NCHS/datalinkage/linked_mortality/

### Random Forest Model Generation, Age Prediction, and Calculation of Age Acceleration

The final 2003–2004 accelerometer data was split into training (70%) and testing (30%) datasets using the caret package in R (Kuhn et al., 2016). A random forest model was generated on centered and scaled data from the training dataset using the randomForest package in R (Breiman and Cutler, 2018). Model parameters were assessed using the randomForest and randomForestExplainer packages in R (Breiman and Cutler, 2018; Paluszynska et al., 2020). The model was validated using the 2005–2006 validation dataset. Finally, to ensure a linear relation between predicted and chronological age, an individual’s predicted age was normalized by dividing by the median predicted ages of individuals of similar chronological ages and multiplying again by the individual’s actual chronological age. Age acceleration and deceleration was then evaluated by subtracting an individual’s chronological age from their normalized biological age prediction. See supplementary materials for extended methods.

### Nutritional and Pharmacological Associations to Biological Age

For correlating nutritional intake to biological age, the 2005–2006 nutrition survey data was downloaded from the NHANES repository and correlations and significant association to deltaAges was assessed using Pearson’s product moment correlation coefficient of binned age groups. Results were clustered using the hclust function in R based on Euclidean distance. Comparisons were further performed between either very high (>10 years) or very low (<10 years) biological age differences (deltaAge) using Student’s t-test. See supplementary materials for extended methods. To screen for drugs that are associated to lower biological aging, the 2005–2006 prescription medication data was downloaded from the NHANES repository and individuals of an advanced age (70–85+). For each drug, the deltaAges of users were compared to the deltaAges of all individuals to identify a significant difference using the non-parametric Kolmogorov-Smirnov test. *p* values were corrected for using the Benjamini and Hochberg method. See supplementary materials for extended methods.

### Statistics

The R programming environment was used for all data processing steps and statistics in this study (R Core Team, 2013). For assessing the random forest model, RMSE was calculated using the Metrics package (Hammer et al., 2018). Pearson’s product moment correlation was used to assess the random forest model and nutritional impact on age acceleration/deceleration. Aging acceleration/deceleration was calculated considering the predicted age minus the normalized biological age. Comparisons of the ratios of accelerated, normal, or decelerated aging relative to mortality was performed using a 3-sample test for equality of proportions without continuity correction. Students t-test was used for two sampled comparisons. The non-parametric test of Kolmogorov-Smirnov was used for comparison of deltaAges of drug users. Throughout this study, *p*-values less than 0.05 were considered significant. When multiple comparisons were performed in large number multiple hypothesis testing corrections were applied to *p*-values using the Benjamini and Hochberg method.

### *C. elegans* Healthspan and Lifespan Measurements

N2 Bristol *C. elegans* were obtained and maintained as previously described (Liu et al., 2020). For mobility crawling measurements, doxazosin mesylate was dissolved in DMSO and added to plates before pouring at a concentration of 33 µM. Worms were synchronized and grown to day 9 or 10 of adulthood. Crawling speed was measured and analyzed as previously described (McIntyre et al., 2021). See supplementary materials for extended methods. Statistical analysis compared treated and untreated conditions using a Mann-Whitney U test. Mobility assays were performed at least twice, one of which is represented in the data shown. Statistics for mobility experiments and replicates are represented in Supplementary Table 3.

Lifespan and motility assays were prepared and analyzed on a microfluidic chip (Infinity Chips, NemaLife Inc., TX, United States) and Infinity Code Software as previously described (Rahman et al., 2020). Doxazosin was tested at 3.3 and 33 μM, as drugs added in microfluidic systems have been shown to act more potently than drugs mixed in agar (Hewitt et al., 2018). Each microfluidics assay was conducted in triplicate (three biological replicates), and each biological replicate consisted of two technical replicates. One technical replicate is a population of ∼60 animals in a microfluidic growth chamber. See supplementary materials for extended methods. Kaplan-Meier curves from the lifespan assays were generated using GraphPad Prism. Log-rank testing was used to compare the survival curves between the non-exposed control and doxazosin-treated populations. Statistics for all lifespan experiments and replicates are represented in Supplementary Table 4. Statistical comparisons of motility were performed in GraphPad Prism using two-way ANOVA. Statistics for motility experiments and replicates are represented in Supplementary Table 5.

## Results

### A Machine Learning Approach to Predict Age From Wearable Device Movement Data

To obtain datasets containing both in-depth characterization of demographic information from individuals and also continuous movement data from a wearable device, we turned to the NHANES data repositories (https://wwwn.cdc.gov/nchs/nhanes/). Data from a single individual can include what drugs they have taken, foods eaten, along with demographic data including age. For earlier survey periods of NHANES, there is also well-documented mortality information for the individuals. The NHANES study periods of 2003–2004 and 2005–2006 are particularly interesting in this regard, as these two study rounds additionally requested individuals to wear a device around their upper thigh measuring their activity levels for a week (ActiGraph AM-7164). Accordingly, we downloaded the accelerometer and demographic data from these study periods, which consisted of several thousands of individuals for each respective study year. We used the 2003–2004 data for model building and testing, then the 2005–2006 data for external validation and exploration of possible effectors influencing the biological aging rate (Figure 1B).

The accelerometer data consisted of a seven-day measurement period with daily entries, and we filtered the data to include only individuals with calibrated instruments that included data for the whole seven days of the study period. Together, this resulted in high quality datasets of 2,634 and 2,505 adults for the 2003–2004 and 2005–2006 datasets, respectively, which covered a broad range of ages from 18 to 85+ (Figure 1C). These data included intensity values at the resolution of minutes for differently aged individuals (Figure 1D), so we aimed to focus the dataset using summary statistics. We reasoned that maximum intensity for each hour could capture elements of vitality in the individual, and the variance of the data for each hour could capture the diversity of movements performed by an individual. Indeed, we found clear differences between these two parameters when assessing different age groups (Figure 1E). This effectively served to reduce our datasets to a summarized form to more efficiently build our MoveAge predictor.

To build the biological age predictor, we split the 2003–2004 dataset into separate training and testing subsets in a 70:30 ratio, and, using the training subset, trained a random forest model to predict an individual’s age from their summarized accelerometer data. We used random forest based on our and others’ previous experiences building age-predictive models (Janssens et al., 2019; Schultz et al., 2020; Shokhirev and Johnson, 2021). Exploring the random forest model’s parameters, we found both maximum activity and variance to have strong predictive abilities for age, with variance throughout the hour having greater influence on the predictive ability of the model (Figure 1F). The time of day also had an influence on the predictor’s strength in the model, with afternoon variance throughout each day of the week having greatest influence (Figure 1F).

Finally, we assessed our model on the 30% validation dataset reserved for this purpose, and found a strong correlation of 0.75 (*p* < 0.01) between predicted and chronological age to exist, with a root mean square error (RMSE) of 13.58 years (Figure 1G). Noting that the model tended to slightly overestimate the ages of younger individuals while underestimating the ages of older individuals, we performed a final normalization with *a priori* knowledge of the chronological age of an individual. Namely, an individual’s predicted biological age was normalized relative to other predicted biological ages of people with the same chronological age. This normalization allowed us to use a more accurate comparison of age acceleration and deceleration per individual, and logically improved our predicted vs. chronological age correlation to 0.94 (*p* < 0.01), with a RMSE of 7.54 years (Figure 1G). As our RMSE values ranged between the RMSE errors of DNA methylation-based epigenetic aging clocks (∼2.9–6 RMSE) (Galkin et al., 2020) and blood-based biomarkers of aging from NHANES (∼14–17.5 RMSE) (Pyrkov and Fedichev, 2019), we reasoned that our model was sufficiently trained to proceed with our study. We termed our final model MoveAge.

### Accelerated Biological Aging From the MoveAge Model Is Linked to Higher Mortality

**FIGURE 2:**
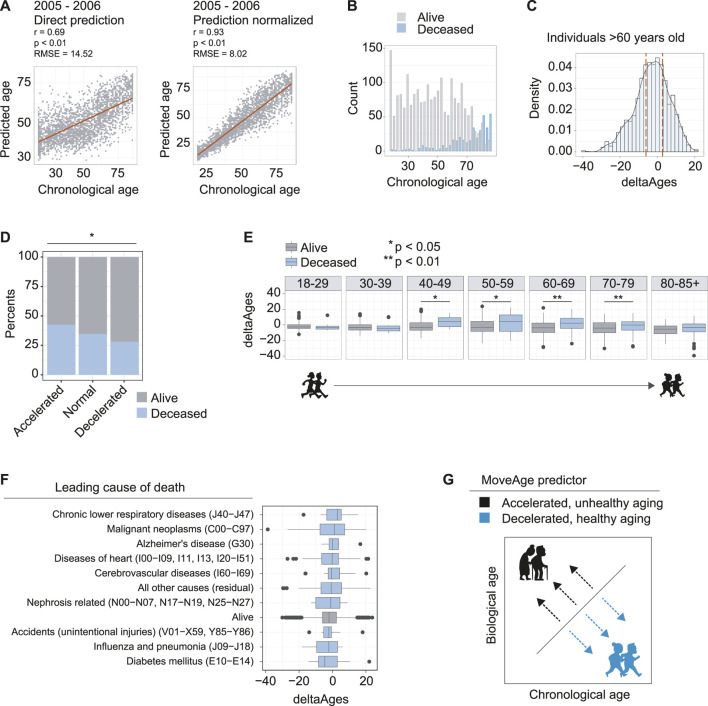
Accelerated biological aging from accelerometer data is linked to higher mortality. **(A)** The model prediction of the 2005–2006 dataset used for validation and exploration. **Left panel** is direct prediction on the raw accelerometer data, *r* = 0.69, *p* < 0.01, and an RMSE of 14.52 years. **Right panel** is the normalization of the prediction based on *a priori* knowledge of the participant’s ages, *r* = 0.93, *p* < 0.01 and an RMSE of 8.02 years. **(B)** Counts of individuals in the 2005–2006 dataset known to be deceased using follow up data from NHANES. **(C)** Distribution of deltaAges of individuals above the age of 60. Calculation of deltaAge is based on the difference between an individual’s predicted biological age and actual chronological age. Dashed lines depict quartiles of the distribution, used to define accelerated, normal, and decelerated aging. **(D)** Ratios of individuals that were alive or deceased in the NHANES follow up assessments, segmented on accelerated, normal, and decelerated aging. Individuals with accelerated aging were more likely to be found deceased, and individuals with decelerated aging were less likely to be found deceased, compared to normal. Significance was calculated using a 3-sample test for equality of proportions without continuity correction *p* < 0.05. **(E)** Comparison of deltaAges for individuals alive or deceased, for each decade of life. Statistics compare deltaAges of the two groups using Student’s t-test *p < 0.05 **p 0.01. **(F)** The deltaAges of individuals alive (grey) relative to top causes of death (blue). Individuals who died from chronic lower respiratory diseases (J40−J47) showed the highest trend towards significantly accelerated aging relative to those who had not died. “Alive” pertains to the NHANES entry presumed alive i.e., alive or no cause of death found. “Nephrosis related” pertains to nephritis, nephrotic syndrome and nephrosis. **(G)** Schematic whereby, relative to chronological age, a higher biological age from the MoveAge predictor corresponds to an accelerated, unhealthy aging phenotype, and a lower biological age corresponds to a decelerated, healthy aging phenotype.

To further validate our model, and explore the relevance of MoveAge, we turned to the 2005–2006 NHANES accelerometer dataset and proceeded to predict the biological ages of individuals based on their movement patterns. Here we confirmed the accuracy of our model, with predicted ages correlating with actual chronological ages at 0.69 (*p* < 0.01), with an RMSE of 14.52 years before normalization. After normalization, our model achieved a correlation of 0.93 (*p* < 0.01) and an RMSE of 8.02 years (Figure 2A).

To further assess how age acceleration or deceleration predicted by our model correlated to biological aging, we related our predictions to the available mortality data within NHANES. Because the 2005–2006 NHANES surveying took place over a decade ago, mortality is recorded in the older age groups of the population (Figure 2B). We then calculated the deltaAge for each individual as the difference between actual chronological age and our predicted biological age, so as to evaluate its relationship to mortality (Supplementary Table 1).

With these elements in hand, we selected the individuals above the age of 60, where mortality becomes more prominent in the data (Figure 2B), and compared the quartile distributions of deltaAges to define groups that had accelerated, normal, or decelerated biological aging (Figure 2C). Comparing the ratio of live to dead from each of the three groups, we found a significant difference whereby individuals with accelerated aging had a greater proportion of mortality (0.47), individuals with decelerated aging had a lower proportion of mortality (0.34) and individuals without altered biological aging had a proportion somewhere in between (0.41) (*p* = 0.018) (Figure 2D). This suggested that our predictions based on movement data captured a biological aging process.

Having observed that higher mortality was present in elderly individuals with accelerated aging, we asked how deltaAge relates to mortality for each decade-sized age bin in our age distributions. For individuals of ages ranging from 18–29, and 30–39, we found no differences in mortality between individuals with age acceleration (Figure 2E). This is likely due to the fact that very few individuals had died in the follow up period, and the few that had were unlikely to be attributable to age-related causes (Figure 2E). However, starting from the 4th decade of life, we found a significant difference, whereby individuals with accelerated aging as predicted from MoveAge had higher incidences of mortality (*p* = 0.011, Figure 2E). This significance was maintained at the 5th (*p* = 0.023), 6th (*p* < 0.01), and 7th (*p* < 0.01) decades of life, with a trend still remaining, though not significant, in the 8th decade (Figure 2E). This suggested that starting from the 40s and proceeding into the next three life decades, accelerated aging plays an increasingly prominent role in determining an individual’s remaining lifespan.

Finally, we asked whether certain causes of death were more linked to accelerated aging as predicted by our model. To address this, we compared the causes of death for each mortality entry to our deltaAge measures. Here we found certain diseases associated with accelerated aging as captured by our model, such as chronic lower respiratory disease, though none were significant with the population size assessed (Figure 2F). Altogether, we conclude that our MoveAge biological age prediction captures the biological aging process in individuals, with higher mortality in individuals with accelerated aging (Figure 2G).

### Identification of Nutritional Components Associated With Decelerated Aging

**FIGURE 3:**
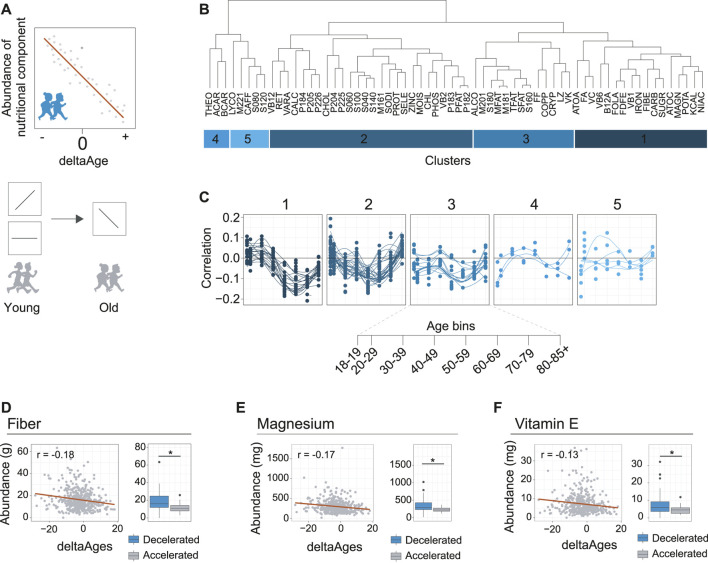
Identification of nutritional components associated with decelerated aging. **(A)** Schematic depicting how nutrient intake may be related to deltaAge. **Upper panel** shows a negative correlation, where increased intake correlates with decelerated aging. **Lower panel** depicts how this trend may change in time. For example, as depicted, a specific nutrient may correlate with accelerated aging, or not correlate with deltaAge, early in life but correlate with decelerated aging later in life. These nutrients could be interesting as potential healthy aging interventions. **(B)** Clustering of nutrients covered in the dietary interview of NHANES based on correlation with deltaAge, assessed in a decade-based manner. This analysis reveals five clusters. Food IDs correspond to the NHANES DR2TOT-D nutrition codes. **(C)** Clusters identified in panel B showing nutritional component abundance correlation with deltaAge across age bins. Cluster one includes nutrient components following the trend that correlates with decelerated aging in the 6th decade of life. **(D)** Fiber, an example of a nutrient from cluster one of panel B. **Left panel** depicts the negative correlation of deltaAge to fiber intake at the 6th decade of life (*r* = −0.18, *p* < 0.01). **Right panel** shows a comparison of fiber intake level of individuals with a deltaAge less than −10 compared to a deltaAge greater than 10, whereby increased fiber intake is associated to decelerated aging (Student’s t-test, *p* < 0.01). **(E)** Magnesium, an example of a nutrient from cluster one of panel B. **Left panel** depicts the negative correlation of deltaAge to magnesium intake at the 6th decade of life (*r* = −0.17, *p* < 0.01). **Right panel** shows a comparison of magnesium intake level of individuals with a deltaAge less than −10 compared to a deltaAge greater than 10, whereby increased magnesium intake is associated to decelerated aging (Student’s t-test, *p* < 0.01). **(F)** Vitamin E, an example of a nutrient from cluster one of panel B. **Left panel** depicts the negative correlation of deltaAge to vitamin E intake at the 6th decade of life (*r* = −0.13, *p* < 0.01). **Right panel** shows a comparison of vitamin E intake level of individuals with a deltaAge less than −10 compared to a deltaAge greater than 10, whereby increased vitamin E intake is associated to decelerated aging (Student’s t-test, *p* < 0.01).

One of the main aims of developing our MoveAge model was to explore nutritional and pharmacological trends that are associated with aging deceleration. To address the nutritional aspect of this, we accessed the dietary intake data available for each NHANES participant. This information is derived from questionnaires that are used to estimate intakes of nutrients, and macromolecule food components. We reasoned that there might be nutritional components whose greater abundance is associated with either aging acceleration or deceleration, and that this could be detected by comparing an individual’s intake to their calculated deltaAge across the population (Figure 3A). Doing so for each decade of life would allow detection of when temporally a food component might affect biological age.

To explore the possibility that dietary components are associated to aging, we proceeded to calculate for each dietary component, at each decade of life, the correlations between deltaAge and the abundance of intake (Supplementary Table 2). We clustered the data to identify patterns occurring across the decades of life (Figure 3B). This revealed how food components had greater or lesser importance for age deceleration. Notably, we found that most food groups had little association with decelerated aging at early ages (Figure 3C). However, a cluster of food components tended to peak after increasing steadily in importance throughout life (Figure 3C, cluster 1). Higher intakes of these specific foods were associated to younger biological ages, growing in importance from the 4th and 5th decade, reaching maximal association at the 6th.

We further explored the components of this cluster, and found the three most significant food components associated with decelerated aging at the 6th decade of life were fiber (r = −0.18, *p* < 0.01), magnesium (r = −0.17, *p* < 0.01), and vitamin E (r = −0.13, *p* < 0.01) (Figures 3D–F, left panels). Higher intake of these foods was associated to decelerated aging. This could further be exemplified by comparing individuals with either very high (>10 years) or very low (<10 years) deltaAges. Doing so revealed a significant association with decelerated aging for all three food components (Figures 3D–F, right panels). Together, these findings are in line with general observations known to benefit health in humans, such as how higher fiber intake is linked to lower mortality (Gopinath et al., 2016), magnesium deficiency is associated to age-related diseases in the elderly (Barbagallo and Dominguez, 2018), and vitamin E may benefit the lifespan of certain human populations (Hemilä and Kaprio, 2011). We therefore conclude that our MoveAge model is useful for the potential identification of factors associated with decelerated aging.

### Retrospective Human *in vivo* Screening for Drugs Associated With Decelerated Aging Identifies Geroprotector Doxazosin

**FIGURE 4:**
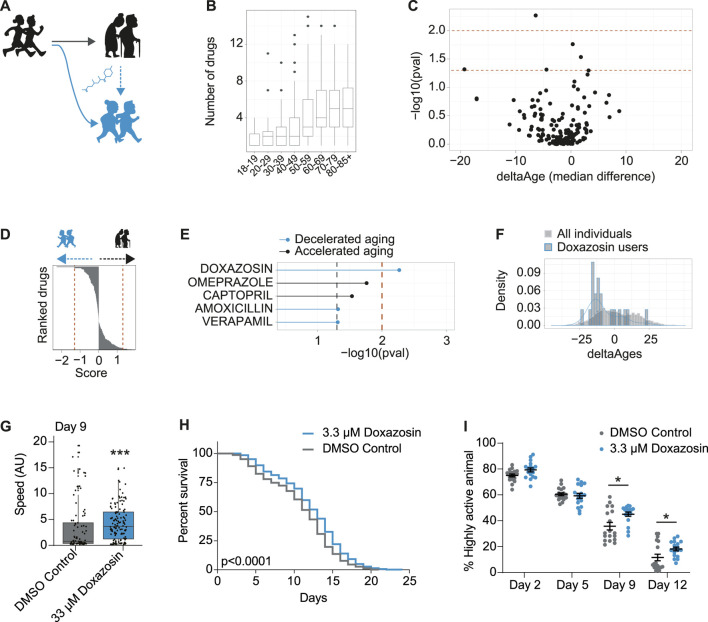
*In vivo* screening in humans for drugs associated with decelerated aging identifies geroprotector doxazosin. **(A)** Schematic to demonstrate use of pharmaceuticals may promote healthy aging or “rejuvenate” from a state of accelerated aging (black) to a state of decelerated aging (blue). **(B)** Number of drugs taken per person, for each decade of life, in the NHANES 2005–2006 dataset. **(C)** Volcano plot including all drugs from all users over the age of 70, whereby the *x*-axis is the median change in deltaAges of users of any given drug, relative to non-users, and the *y*-axis is the significance of this difference (−log10 *p*-value, Kolmogorov-Smirnov test). Dashed lines correspond to *p* < 0.05 and *p* < 0.01. **(D)** Based on panel C, drugs ranked by their decelerating or accelerating effect on aging. Rank is given to each drug by multiplying their log10 *p* value by the sign of deltaAge (negative or positive). **(E)** The top compounds from panels C and D associated with either accelerated or decelerated aging. Only one compound is significant at *p* < 0.01: doxazosin. **(F)** The distribution of deltaAges of doxazosin users relative to all individuals (depicting top result from panel C). Density plot overlaid on top of histogram (*n* = 10 for doxazosin and 1922 for all other drugs). **(G)** Mobility as determined by crawling speed on day 9 of life, for *C. elegans* grown on DMSO control (0.2%), or 33 μM doxazosin. Worms treated with doxazosin are significantly more mobile than untreated (*n* = ∼80–130 worms per condition, *p* < 0.001, Mann-Whitney U Test). Replicates and statistics of mobility experiments can be found in Supplementary Table 3. **(H)** Survival curves showing that 3.3 μM doxazosin, extends lifespan in wild type (N2) *C. elegans* (*n* = ∼450 worms per condition, *p* < 0.0001, Log-rank test). Results are pooled from three independent experiments. Individual replicates and statistics can be found in Supplementary Table 4. **(I)** Worm activity healthspan as measured by motility on days 2, 5, 9, and 12 of adulthood. On days 9 and 12, the percentage of highly active worms is significantly higher for the population treated with 3.3 μM doxazosin (*n* = 18 videos analyzed per condition, *p* < 0.05, Two-way ANOVA). Results represent pooled data from three independent experiments. Individual replicates and statistics of motility experiments can be found in Supplementary Table 5.

Our final aim was to identify if specific drugs that individuals in our dataset were taking may also be associated to decelerated aging. This approach would, in effect, constitute a human *in vivo* screening for compounds promoting healthy aging. While false positives may result from this approach in the form of compounds only associated, rather than causing, age deceleration (e.g., antibiotics or antihistamines, which are the main drugs that otherwise healthy individuals may take), it could nonetheless reveal compounds that contribute to healthy aging (Figure 4A).

To identify compounds associated to decelerated aging, we focused on the 70+ population who was both elderly and most likely to be taking pharmaceuticals (Figure 4B). Then, for each drug, we compared the distribution of deltaAges of users of that drug, relative to the distribution of deltaAges of others in this same demographic. This allowed us to generate both a significance level comparing these distributions, and a median fold change in delta ages that could be represented as a volcano plot (Figure 4C). With these metrics we could rank the compounds based on their *p*-values (Figure 4D), and, a non-stringent significance criteria (*p* < 0.05) could generate a list of compounds that were most associated with decelerated aging (doxazosin, amoxicillin, verapamil) or accelerated aging (omerprazole, captopril) (Figure 4E). While no drug of the 500 + FDA approved drugs we assessed passed significance following a correction for multiple hypothesis testing (Benjamini and Hochberg), one compound—doxazosin—passed significance using a stringent unadjusted *p*-value cutoff (*p* < 0.01) (Figure 4E). Users of doxazosin were more likely to have negative deltaAges, representing a possible age-deceleration due to the drug, when compared to all others in the same demographic population (Figure 4F).

As stated above, the association between doxazosin and decelerated age does not necessarily mean it causally benefits healthy aging. We therefore turned to *C. elegans* to test if doxazosin was causally geroprotective. We used *C. elegans* because it is a well-described aging model organism, and has been used to identify and understand other lifespan extending drugs, such as metformin and others (Cabreiro et al., 2013; Ye et al., 2014). We based our original screen on movement data, so we aimed to determine if doxazosin could improve the worms’ healthspan, which we initially tested using a measure of age-related mobility later in life. When worms were grown on agar plates that contained 33 μM doxazosin, a dose previously shown to extend lifespan in worms (Ye et al., 2014), they crawled across the plate at significantly higher speeds than those treated with the vehicle at day 9 or 10 of adulthood (*p* < 0.001) (Figure 4G; Supplementary Table 3). We then used a microfluidics platform totest lifespan and healthspan. Here we used multiple doses of doxazosin (3.3 and 33 μM) as previous observations show that microfluidics devices provide more direct drug contact than agar plates, and therefore a lower dose is needed (Hewitt et al., 2018). Indeed, we saw the greatest beneficial effects at 3.3 μM (Figures 4H,I; Supplementary Tables 4, 5). We confirmed that doxazosin extended worm lifespan (*p* < 0.0001) (Figure 4H; Supplementary Table 4) and simultaneously studied worm motility so as to measure healthspan *via* locomotion. With automated locomotion measurements, we calculated what percentage of worms remained highly active (defined as moving a distance greater than their body length in a 30 s window). Upon doxazosin treatment, we observed a higher percentage of highly active animals throughout life, particularly in the later ages tested, confirming their improved healthspan (Figure 4E; Supplementary Table 5). Altogether, we conclude that doxazosin extends both lifespan and healthspan in *C. elegans*.

## Discussion

In this study, we built a model to predict human biological age based on movement data available from a wearable device. The difference between chronological age and the predicted age produced by our model (deltaAge) allowed us to define an individual’s aging as decelerated (healthy aging), normal, or accelerated (unhealthy aging), and accelerated aging in middle-to older-age populations was significantly associated with mortality. Using this model, we searched for nutritional components and pharmaceutical drugs associated with decelerated aging. We identified a group of nutritional components, and one pharmaceutical: doxazosin. We causally connected doxazosin to healthspan and longevity in *C. elegans* by demonstrating that doxazosin treatment extends age-related movement ability and lifespan in worms.

Our model, MoveAge, was built using NHANES data on movement patterns over one week, which we summarized to hourly maximum intensity and hourly variance. One logical drawback of this approach is that our model could be capturing patterns of life, rather than biological vitality. Activity in the afternoon and evening had the strongest predictive power for the model, which, for example, could be related to an individual’s work schedule rather than their health. Additionally, our model may not capture all aspects of declining health occurring during aging, such as increased incidence of cancer or mental decline. However, our model, like the other models produced using movement data to predict relative physical mobility as a measure of biological age (Pyrkov et al., 2018a; Pyrkov et al., 2019; Rahman and Adjeroh, 2019), is associated with mortality in the population we tested. Those with high deltaAges were significantly more likely to be deceased in the follow-up data available through NHANES. Therefore, we conclude that our model indeed captures aspects of biological vitality in the predicted ages produced, which may also correlate with lifestyle patterns.

After confirming that deltaAges from MoveAge associated with mortality, we determined if any specific causes of death were connected to accelerated aging. We found that of all the causes of death recorded by NHANES, chronic lower respiratory diseases was the most associated with increased deltaAge. This could potentially be due to a reduction in the capacity to exercise or move brought on by shortness of breath in these sorts of diseases. As some of the most severe repercussions of COVID-19 infection are respiratory complications (Hu et al., 2021), biological age could be useful as a predictor for those most severely affected by the disease (Pyrkov et al., 2021).

Our work builds on previous studies that have developed biological age predictive models from wearable device movement data (Pyrkov et al., 2018b; Pyrkov et al., 2019; Rahman and Adjeroh, 2019). We expand on this theme by utilizing MoveAge to identify nutritional components and pharmaceuticals that are associated with decelerated aging, and therefore could play a geroprotective role. The nutritional components we identify associated with healthy aging, but further research is necessary to determine their causal role in aging deceleration. Even so, the increased significance of such components later in age leads us to speculate about the possibility of personalized nutrition advice to promote healthy aging. Perhaps if an individual has signs of accelerated aging, addition of these sorts of nutritional components could act as a prophylactic treatment, slowing health degeneration.

In contrast to the nutritional components identified, we not only associate doxazosin with decelerated aging, but also demonstrate its causal role in longevity in *C. elegans*. Lifespan extension in worms treated with doxazosin has been previously described in a high throughput screen (Ye et al., 2014), and our work confirms this in a high resolution microfluidic lifespan assay. We additionally demonstrate doxazosin’s significant benefits for healthspan, using both crawling mobility and motility assays, which we considered highly relevant as MoveAge is based on age-based movement. These two healthspan assays occur in distinct environments, suggesting a robust effect. We also acknowledge that, while we do show that doxazosin extends lifespan and healthspan in worms, further study is necessary to confirm its causal geroprotective effects in humans.

As an alpha-adrenergic blocker, doxazosin inhibits the activation of post-synaptic alpha-1 receptors by norepinephrine, thereby opposing blood vessel contraction (Taylor, 1989). It is still unclear how inhibition of the alpha-adrenergic receptor (*ADRA2A* in humans) may influence lifespan in worms. Suppression of the gene orthologous to *ADRA2A* in worms, *octr-1*, increased the generalized unfolded protein response (UPR) (Y. Liu et al., 2016) and the UPR^ER^ (Özbey et al., 2020), and activation of both the UPR and UPR^ER^ are associated with extended lifespan in worms (Taylor and Dillin, 2013; Janssens et al., 2019). Additionally, in the original screen that identified doxazosin’s lifespan extension capability, multiple other drugs targeting adrenoreceptors were identified to extend lifespan (Ye et al., 2014). Lifespan effects and oxidative stress were also significantly correlated in this class of drugs (Ye et al., 2014). Taken together, these findings suggest that the unfolded protein response and oxidative stress resistance pathways could be interesting initial routes of investigation to understand doxazosin’s mechanism of action. This could in turn provide deeper insight into aging pathways and points of more directed intervention.

In conclusion, our results confirm recent studies demonstrating the capacity to accurately predict biological age from accelerometer-based movement data, and provide an additional biological age prediction model to the field. We further build on existing work to demonstrate the capacity to use such models to identify the geroprotective capacity of lifestyle factors or pharmaceuticals in the population studied. Through such analysis, we associate fiber, magnesium and vitamin E with longevity, as well as the alpha-blocker doxazosin. We further confirm doxazosin as a geroprotective compound, and in doing so, demonstrate the power of using biological age predictors as *in vivo* screening tools.

## Data Availability

The original contributions presented in the study are included in the article/Supplementary Material, further inquiries can be directed to the corresponding author.
