# Supplementary material for: Biological Age Prediction From Wearable Device Movement Data Identifies Nutritional and Pharmacological Interventions for Healthy Aging
Source: Front Aging. 2021 Jul 15;2:708680. doi: 10.3389/fragi.2021.708680 (PMC9261299; doi:10.3389/fragi.2021.708680)
Supplement: Supplementary file 1 [file DataSheet1.zip › Supplementary Materials.pdf]

Extended methods:

Accelerometer data processing

The 2003-2004 accelerometer data was downloaded from the NHANES repository and included entries for 7176 individuals. Data was initially filtered to ensure that data was present for each minute of the seven days (an entry was allowed to be '0') and that devices were calibrated (using the PAXCAL data column provided by NHANES). This resulted in data for 6790 individuals. Furthermore, data was filtered to ensure that seven days of entries were present per individual. A day was considered to contain sufficient entries if more than 10% of the day included accelerometer data with a value greater than 0. Finally, data was further filtered to only include adults (aged 18 and older). These filtering steps to ensure high quality data resulted in a final dataset size of 7 days of accelerometer data for 2634 adults. For each individual, data was summarized on an hourly basis by considering the maximum intensity value of each hour and the variance of the data in that hour. The 2005-2006 NHANES accelerometer data was downloaded and preprocessed identically to the 2003-2004 data, which resulted in a final validation dataset with accelerometer data for 2505 adults.

Random Forest model generation and age prediction

The final filtered, summarized data from 2003-2004 of NHANES was split into training (70%) and testing (30%) datasets using the caret package in R (Kuhn et al., 2016). The training dataset was used to generate scaling and centering parameters to the data using the preprocess function in the caret package in R. A random forest model was generated on the centered and scaled data from the training dataset using the randomForest function in the randomForest package in R (Breiman & Cutler, 2018). All parameters were kept as default and no manual tuning or optimization was performed on the model. For steps involving random number generation, seed was set to the '2019' (the year this study was started). Model parameters were assessed using the randomForest and randomForestExplainer packages in R (Breiman & Cutler, 2018; Paluszynska, Biecek, & Jiang, 2020). The model was validated using the 2005-2006 validation dataset with centering and scaling preprocessing parameters derived from the 2003-2004 training data. After the model made predictions for biological age based on accelerometer data, these predictions were normalized based on the biological age predictions of an individual's peers and their own chronological age. Specifically, an individual's predicted age was normalized by dividing by the median predicted ages of individuals of similar chronological ages (grouped by 5 year increments), and multiplying again by the individual's actual chronological age.

Nutritional data processing

Total nutrient intakes as calculated by NHANES on the first day of survey (DR1TOT_D) were used for comparison to deltaAges of individuals. Pearson's correlations between deltaAge and intake abundance for each nutrient at each decade of life were calculated. Results were treated as a time series and clustered using the hclust function in R based on Euclidean distance. Upon visual inspection of the dendrogram, five clusters were selected for further assessment. Within these, food components with greatest correlation or anticorrelation to deltaAge, and possessing the lowest p-value were considered. Correlations and significance were tested for using Pearson's product moment correlation coefficient. In comparing the distribution of a nutrient's abundance in individuals with either very high (>10 years) or very low (<10 years) biological age differences (deltaAge), Student's t-test was used for significance. Food component definitions were accessed through
https://wwwn.cdc.gov/Nchs/Nhanes/2005-2006/DR1TOT_D.htm

Drug data processing

Prescription medication data for the 2005-2006 NHANES cohort was downloaded from the NHANES website. Prescription medication for individuals of an advanced age (70-85+ years) was used to assess the relationship between biological age and drug intake. For each drug, the distribution of deltaAges of all of its users was compared to the distribution of deltaAges of all of the nonusers within the same age demographic using the non-parametric Kolmogorov-Smirnov test. Values were corrected for using the Benjamini & Hochberg method. Compounds were ranked based on p-value and whether they accelerated (positive sign) or decelerated (negative sign) biological aging. Prescription medication definitions were accessed through:
https://wwwn.cdc.gov/Nchs/Nhanes/2005-2006/RXQ_RX_D.htm

***C. elegans* maintenance**

C. elegans strains N2 Bristol and *E. coli* strain OP50 were obtained from *Caenorhabditis* Genetics Center (CGC; University of Minnesota, Minneapolis, USA). Hermaphrodite worms were grown and maintained on nematode growth media (NGM) agar plates seeded with OP50 *E. coli* at 20 °C as previously described (Liu et al., 2020).

Worm mobility measurements

Doxazosin mesylate was obtained from Sigma Aldrich and dissolved in DMSO at a concentration of 33 mM. The compound was added to NGM agar plates just before pouring at the concentrations described. Gravid adult worms were age-synchronized using alkaline hypochlorite treatment, and incubated in M9 buffer overnight. L1 stage worms were seeded to NGM plates. Worms were transferred to plates supplemented with 33 µM doxazosin and 10 µM 5-fluorouracil (Sigma Aldrich) at the L4 larval stage. All assays were performed at 20°C,

and the L4 stage was counted as day 0 of adulthood. Plates were changed once per week to maintain exposure to the compounds.

At day 9 or 10 of adulthood, ~50 worms were transferred to NGM plates without OP50, stimulated by tapping the plate, and immediately recorded for 200 cycles at room temperature using a Leica (Amsterdam, The Netherlands) M205 FA fluorescent microscope and Leica DFC 365 FX camera. Images were captured using Leica Application Suite X software, then processed with the wrMTrck plugin for ImageJ (Nussbaum-Krammer et al., 2015). Data from wrMTrck were analyzed and visualized using a custom script in R (R Core Team, 2013). Statistical analysis compared treated and untreated conditions using a Mann-Whitney U test. Mobility assays were performed at least twice, one of which is represented in the data shown. Statistics for mobility experiments and replicates are represented in Table S3.

Microfluidics survival and motility assays

Wild type (N2) *C. elegans* were cultured on 60 mm petri dishes (Fisher Scientific; Austin, TX, USA) on a standard food source of *E. coli* OP50 and incubated for 48 hours at 20°C. For age synchronization, a suspension of gravid adults in 20 mg/mL *E. coli* OP50 were loaded into microfluidic chips (Rahman et al., 2020) (Infinity Chips, NemaLife Inc., TX, USA) and allowed to lay eggs for 2 hours. These progeny were grown for 3 days and the loaded into microfluidic chips along with 20 mg/mL of *E. coli* OP50 in liquid NGM. Several concentrations of doxazosin were formulated in liquid NGM and mixed with DMSO (Fisher Scientific). In all tested doxazosin solutions, the final concentration of DMSO was maintained at 0.2 % v/v and the food concentration was maintained at 20 mg/mL of *E. coli* OP50.

Each microfluidics assay was conducted in triplicate (three biological replicates), and each biological replicate consisted of 2 technical replicates. One technical replicate is a population of ~60 animals in a microfluidic growth chamber.

For each lifelong assay, videos were acquired each day to determine live counts, prior to feeding fresh doxazosin solutions. L4 stage was counted as day 0 of adulthood. Videos were analyzed using the Infinity Code software (NemaLife Inc., TX) for animal survival and motility. The number of living animals in the population was determined based on detectable movement. Motility was determined based on the displacement of individual animals from the rectangular area (bounding box) that encloses their whole body. Animals that moved more than their body length within 30 seconds were labelled “highly active.” The percentage of highly active animals in the population was then calculated. Statistical comparisons were performed in GraphPad Prism using two-way ANOVA. Statistics for motility experiments and

replicates are represented in Table S5. Kaplan-Meier curves from the lifespan assays were generated using GraphPad Prism. Log-rank test was used to compare the survival curves between the non-exposed control and doxazosin-treated populations. Statistics for all lifespan experiments and replicates are represented in Table S4.

References:

Breiman, L., & Cutler, A. (2018). *randomForest: Breiman and Cutler's Random Forests for Classification and Regression*. Retrieved from https://www.stat.berkeley.edu/~breiman/RandomForests/

Kuhn, M., Wing, J., Weston, S., Williams, A., Keefer, C., Engelhardt, A., … Hunt, T. (2016). *Caret: Classification and Regression Training*. Retrieved from https://cran.r-project.org/package=caret

Liu, Y. J., McIntyre, R. L., Janssens, G. E., Williams, E. G., Lan, J., van Weeghel, M., … Houtkooper, R. H. (2020). Mitochondrial translation and dynamics synergistically extend lifespan in C. elegans through HLH-30. *The Journal of Cell Biology*, *219*(6). https://doi.org/10.1083/jcb.201907067

Nussbaum-Krammer, C. I., Neto, M. F., Brielmann, R. M., Pedersen, J. S., & Morimoto, R. I. (2015). Investigating the spreading and toxicity of prion-like proteins using the metazoan model organism C. elegans. *Journal of Visualized Experiments : JoVE*, (95), 52321. https://doi.org/10.3791/52321

Paluszynska, A., Biecek, P., & Jiang, Y. (2020). *randomForestExplainer: Explaining and Visualizing Random Forests in Terms of Variable Importance*. https://doi.org/10.1198/jasa.2009.tm08622

R Core Team. (2013). *R: A language and environment for statistical computing.* Retrieved from http://www.r-project.org/

Rahman, M., Edwards, H., Birze, N., Gabrilska, R., Rumbaugh, K. P., Blawzdziewicz, J., … Vanapalli, S. A. (2020). NemaLife chip: a micropillar-based microfluidic culture device optimized for aging studies in crawling C. elegans. *Scientific Reports*, *10*(1), 1–19. https://doi.org/10.1038/s41598-020-73002-6

Supplemental tables:

Table S1. Predicted ages of individuals in validation/exploration (2005-2006) dataset

Table S2. Dietary component correlations with deltaAges

Table S3. Worm mobility replicates and statistics

***C. elegans* strain**	**Treatment**	**Average speed**	**Standard Deviation**	**Number animals**	**P – value against control group**
Wild Type (N2)*	DMSO control (0.2%)	3.5026	4.8514	89	
	33 μM doxazosin	4.3526	3.4115	132	0.0006
Wild Type (N2)	DMSO control (0.2%)	5.1146	5.2565	93	
	33 μM doxazosin	6.2725	3.6412	83	0.0024

*denotes replicate represented in figure

Table S4. Worm lifespan replicates and statistics

C. elegans strain	Treatment	Median lifespan (days)	% Change	Number animals (died/total)	P – value against control group
Wild Type (N2)*	DMSO control (0.2%)	12		442/448	
	3.3 μM doxazosin	13	8.33%	425/462	<0.0001
	33 μM doxazosin	12	0.00%	427/435	0.0081
Wild Type (N2)	DMSO control (0.2%)	13		143/145	
	3.3 μM doxazosin	14	7.69%	139/145	0.1506
	33 μM doxazosin	12	-8.33%	144/145	0.2916
Wild Type (N2)	DMSO control (0.2%)	10		146/146	
	3.3 μM doxazosin	12	20.00%	143/166	0.0003
	33 μM doxazosin	12	20.00%	137/137	0.0002
Wild Type (N2)	DMSO control (0.2%)	12		153/157	
	3.3 μM doxazosin	13	8.33%	143/151	0.0012
	33 μM doxazosin	12	0.00%	146/153	0.0145

*denotes pooled results represented in figure of the three below individual replicates

Table S5. Worm motility replicates and statistics

**Day 2**					
***C. elegans* strain**	**Treatment**	**Average % highly active**	**Standard deviation**	**Number videos**	**P – value against control group**
Wild Type (N2)*	DMSO control (0.2%)	74.125	4.616	18	
	3.3 μM doxazosin	78.363	6.22	18	0.2627
	33 μM doxazosin	76.340	9.427	18	0.6920
Wild Type (N2)	DMSO control (0.2%)	76.150	3.653	6	
	3.3 μM doxazosin	78.117	9.581	6	0.8825
	33 μM doxazosin	71.317	14.405	6	0.4747
Wild Type (N2)	DMSO control (0.2%)	74.283	4.111	6	
	3.3 μM doxazosin	78.573	5.626	6	0.2027
	33 μM doxazosin	81.886	3.099	6	0.0090
Wild Type (N2)	DMSO control (0.2%)	71.941	5.628	6	
	3.3 μM doxazosin	78.400	2.997	6	0.0566
	33 μM doxazosin	75.817	4.181	6	0.3419
**Day 5**					
***C. elegans* strain**	**Treatment**	**Average % highly active**	**Standard deviation**	**Number videos**	**P – value against control group**
Wild Type (N2)*	DMSO control (0.2%)	59.791	4.767	18	
	3.3 μM doxazosin	58.539	8.056	17	0.8919

	33 μM doxazosin	56.402	9.754	18	0.4241
Wild Type (N2)	DMSO control (0.2%)	60.967	7.471	6	
	3.3 μM doxazosin	56.757	6.232	6	0.5685
	33 μM doxazosin	60.867	8.380	6	0.9997
Wild Type (N2)	DMSO control (0.2%)	59.333	3.087	6	
	3.3 μM doxazosin	65.427	5.388	6	0.0441
	33 μM doxazosin	61.840	8.094	6	0.5733
Wild Type (N2)	DMSO control (0.2%)	59.072	5.628	6	
	3.3 μM doxazosin	52.400	7.197	5	0.0614
	33 μM doxazosin	46.500	3.271	6	<0.0001

Day 9

***C. elegans* strain**	**Treatment**	**Average % highly active**	**Standard deviation**	**Number videos**	**P – value against control group**
Wild Type (N2)*	DMSO control (0.2%)	35.316	12.142	18	
	3.3 μM doxazosin	44.490	6.823	18	0.0024
	33 μM doxazosin	44.493	7.504	18	0.0024
Wild Type (N2)	DMSO control (0.2%)	50.733	4.558	6	
	3.3 μM doxazosin	47.433	1.143	6	0.7046
	33 μM doxazosin	53.250	4.538	6	0.8152
Wild Type (N2)	DMSO control (0.2%)	25.467	3.772	6	
	3.3 μM doxazosin	47.204	2.893	6	<0.0001
	33 μM doxazosin	41.730	3.880	6	<0.0001
Wild Type (N2)	DMSO control (0.2%)	29.747	5.234	6	
	3.3 μM doxazosin	38.833	9.538	6	0.0045
	33 μM doxazosin	38.500	3.391	6	0.0064

Day 12

***C. elegans* strain**	**Treatment**	**Average % highly active**	**Standard deviation**	**Number videos**	**P – value against control group**
Wild Type (N2)*	DMSO control (0.2%)	11.375	10.774	18	
	3.3 μM doxazosin	44.490	5.884	18	0.0457
	33 μM doxazosin	20.987	7.621	18	0.0014
Wild Type (N2)	DMSO control (0.2%)	25.733	3.995	6	
	3.3 μM doxazosin	18.500	2.958	6	0.1943
	33 μM doxazosin	28.000	8.250	6	0.8472
Wild Type (N2)	DMSO control (0.2%)	4.383	2.571	6	
	3.3 μM doxazosin	23.590	3.008	6	<0.0001
	33 μM doxazosin	13.962	2.444	6	0.0008
Wild Type (N2)	DMSO control (0.2%)	4.008	0.953	6	
	3.3 μM doxazosin	11.500	3.391	6	0.0225
	33 μM doxazosin	21.000	2.280	6	<0.0001

*denotes pooled results represented in figure of the three below individual replicates
